# Tuned by time: the role of circadian rhythms in metabolic energy sensing and chronotherapy

**DOI:** 10.1080/07853890.2025.2596548

**Published:** 2025-12-03

**Authors:** Arun Thanuskodi Rajakumar, Gothandam Kodiveri Muthukaliannan

**Affiliations:** Department of Biotechnology, School of Bio Science and Technology, Vellore Institute of Technology, Vellore, Tamil Nadu, India

**Keywords:** Circadian rhythm, feeding pattern, metabolism, AMPK, sirtuins, energy signalling, chronotherapy

## Abstract

**Introduction:**

Circadian rhythms are produced endogenously from oscillators that are based on transcription-translation feedback loops, which can synchronize central and peripheral clocks into orderly timing of physiological processes to ensure the precision of metabolic homeostasis in organ such as liver, muscle, pancreas and adipose tissue. The master circadian clock located in the suprachiasmatic nucleus (SCN) of the anterior hypothalamus receives light signals and synchronizes the other internal clocks in important metabolic organs. Nuclear receptors regulate the circadian secretion of hormones and AMP-activated protein kinase (AMPK) and Sirtuins (SIRTs) represent favoured energy-sensing mechanisms in these tissue-specific clocks. This review summarizes the interplay between circadian rhythms and metabolism as well how a restoration of the circadian mechanism with the use of targeted treatments benefiting the metabolic dysfunction of a world with lengthened light exposure.

**Discussion:**

Lifestyle habits of contemporary life such as artificial light at night (ALAN) and irregular feeding regime disturb these important oscillations and interfere with physiological function and metabolism. As a result, peripheral clocks become decoupled from light-dark cycle and influence glucose absorption into the muscles. Among the disruptions, the underlying molecular mechanism includes intricate interactions among core clock genes (CLOCK, BMAL1, CRY, PER), regulation of the NAD+/NADH ratio and the lactate shuttle. Time is therefore a critical variable in the context of homeostasis as witnessed by such processes as circadian entrainment and metabolic flux. Novel chrono therapeutics are required to restore the circadian homeostasis, including time-restricted feeding, light and pharmacotherapy that responds to the circadian rhythm, in the aim of improving metabolic parameters.

**Conclusion:**

Findings suggest that circadian disruption has a significant causal role in the development of metabolic disease in modern civilization. Chronotherapeutic strategies to enhance therapeutic efficacy and decrease adverse reactions by aligning treatment to an individual’s time of circadian sensitivity represent one approach for restoring circadian homeostasis to remedy the ills of the modern illuminated world.

## 1. Introduction

A fundamental adaptation mechanism, where plants open stomata during the day and close them at night, which allows them to synchronize with the 24-hour day-night cycle on Earth, thus maximizing photosynthesis and the efficiency of water use [1]. The circadian rhythms produced endogenously are represented by the ∼24-hour oscillations that are maintained by transcription-translation feedback loops (TTFLs) that regulate the patterns of gene expression in line with the environmental cues (i.e. light, temperature and so forth). Located at the very top of this hierarchy is the suprachiasmatic nucleus (SCN), a bilateral structure in the anterior hypothalamus, and that serves as the primary circadian pacemaker [2]. It receives photic input using intrinsically photosensitive retinal ganglion cells containing melanopsin. Consequently light is conveyed back to SCN through retinohypothalamic allowing its entrainment to environmental cues [3]. Clock genes coordinate the molecular oscillations in SCN, which in turn harmonize the downstream neuron and hormone processes [4]. The central circadian pacemaker disseminates time cues to peripheral oscillators in tissues of adipose, liver, as well as skeletal muscle through neural, endocrine and behavioural pathways [5].

Peripheral oscillators that are found within specific tissues have tissue-specific rhythmic gene expression profiles, which are essential in maintaining homeostasis [6]. The liver circadian regulation modulates gluconeogenesis, glycogenesis and lipid metabolism through the harmonization of major metabolic genes [7]. The fatigue of these rhythms is commonly instigated by the lifestyle factors like Artificial Light at Night (ALAN) that had been aggravated because of growing industrialization and digitalization, rigidly establishing a 24/7 work culture leading to metabolic dysregulation and pathophysiological conditions. In addition to that, the increase in the number of night shifts employed is directly proportional to the reported increase in the EU (19%) and the United States (26%) [8], ALAN pollution which is increasing by about 10% annually [9]. This rising exposure has a severe effect on SCN-mediated melatonin production with the highest suppression being under short wavelength light (∼459 nm) especially the blue light. Thereby affecting its natural nighttime peak under light-dark conditions which coincides with sleep onset and regulates the sleep-wake cycle as well as antioxidant defence function governed by the pineal gland [10]. Nevertheless, the melatonin inhibition caused by ALAN does not only impair sleep, it also decreases the availability of serotonin an essential precursor to both anabolic and catabolic pathways impairing metabolic equilibrium [11]. Also, SCN failure to keep track with peripheral clocks exacerbates the dysregulation of insulin sensitivity and lipids profiles thus contributing obesity, T2DM and non-alcoholic fatty liver disease [12].

Abnormal feeding routines lead to pathological circumstances concerning glucose metabolism in the liver which produce chronic inflammatory phenomena and oxidative pressure [13]. Studies of the diurnal expression patterns of mouse nuclear receptors in the principle lipid and glucose metabolism organs indicate extensive expression in the organs and tissue-specific fluctuations [14]. The nuclear receptors assist in the appropriate regulation of lipid and glucose level by transcriptional and post-transcriptional processes. The Suprachiasmatic Nucleus may be an important communicator of metabolic circuitry *via* projections in particular to the Arcuate Nucleus (ARV) and Paraventricular Nucleus (PVN) which control hunger and hepatic glucose production [15]. This indicates that food intake patterns are extremely important as one of the most significant Zeitgeber (ZT) for peripheral clocks particularly in metabolic tissues such as liver. SCN ablations indicate that food timing alone does not function as a dominant Zeitgeber highlighting the SCN’s critical role for peripheral signalling pathways and emergent circadian rhythms [16].

Disruption of these regulatory pathways produced *via* decremented dark conditions or prolonged light exposures in nocturnal conditions produces negative metabolic effects. Factors such as lack of physical activity, poor dietary habits, along with long-term disruption of the body’s innate circadian rhythm create a situation of metabolic imbalance, also by causing maladaptive mitochondrial dysfunction [17]. This ill effect appears in the circadian regulation of the functionality of adipose tissue, which suggests that white and brown adipocytes are subject to circadian patterns in gene expression affecting lipid storage, thermogenic response to drugs and circulating hormone signals affecting adipokine secretion [18]. Regulatory mechanisms between these connections are strictly controlled by SCN-mediated signals that coordinate peripheral clocks *via* pre-autonomic neurons, modulating hepatic glucose by acting on central and peripheral targets to influence energy homeostasis [19,20]. This review therefore presents some mechanistic details of how transcription factors related to circadian rhythms operate and some of their functional features in the establishment of metabolic health, all in the context of the disruption of circadian rhythms which is so prevalent in present-day lifestyles.

## 2. Circadian rhythms

The three important parameters of circadian rhythms are: Amplitude, representing difference in peak and trough levels, such as in hormone or activity levels; Phase, indicating the timing of the occurrence of reference point within cycle, such as peak relative to night onset; and Period, which is the time elapsed between recurring points, such as two consecutive peaks [21]. These rhythms continue even when light is continuous or absent; this phenomenon is termed ‘free running’. Light exerts a potent effect on the resetting of rhythm through phase shifts, either by delaying or advancing the cycle based on exposure timing. Early subjective nighttime light exposure causes phase delay, while late subjective nighttime light exposure causes phase advance [22]. It results into a small or no change in the exposure of light during the subjective day showing its phase-dependent resetting effect. This reciprocal process guarantees that the biological clock of the organism is in balance with the light-dark cycles of the environment.

Cyclic alterations in the expression of certain genes may be the cause of the activation of the internal pacemaker, as the oscillations progress. The identity and function of each tissue are determined by the expression of tissue-specific genes, as instructed by the epigenetic code [23]. Likewise, circadian code controls timing of gene expression within each tissue. Orchestrating daily rhythms that contribute to diurnal patterns in physiology, metabolism and behaviour [24]. Proteomics study on various species shows that proteins exhibit rhythmic fluctuations driven by circadian genes, supporting the theory that gene expression and protein production are crucial for normal clock function [25]. These dynamic changes are regulated through interlocking transcriptional-translational feedback loops, which operate across distinct phases of the circadian cycle, known as Zeitgeber Time (ZT).

**Figure 1.**
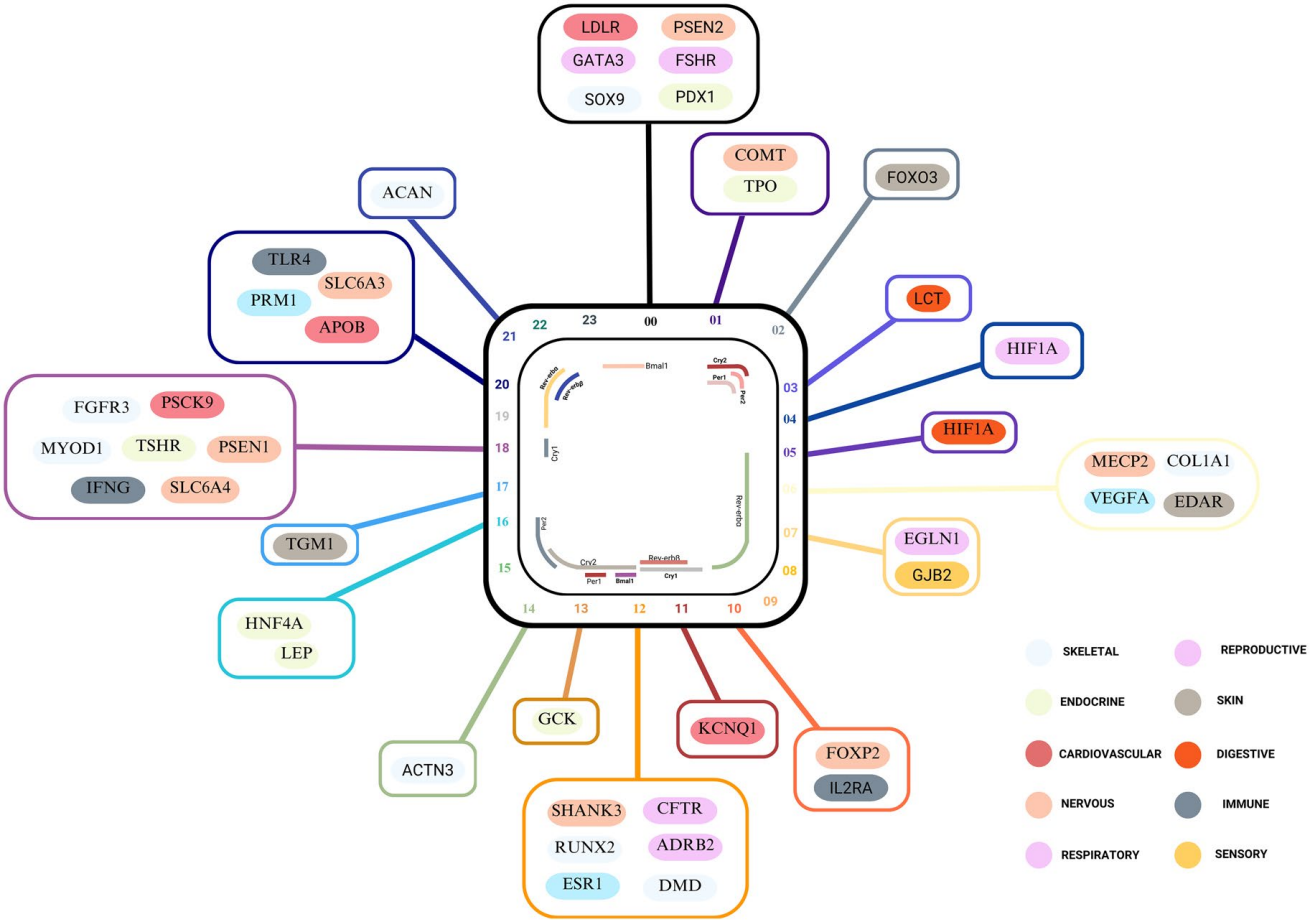
Illustrates the synchronization between key circadian clock genes in human (light) and mouse (dark) and their associated physiological systems. The central region represents the acrophase of core circadian-regulated genes, while the branched coloured lines and rectangles denote the timing of peak protein expression in peripheral organ systems. Each system is colour-coded by function: skeletal, respiratory, endocrine, reproductive, digestive, cardiovascular, immune and neurophysiological. This temporal alignment between their expression and physiological activity underscores the phase relationship between central and peripheral oscillators in maintaining systemic homeostasis.

The oscillator, outputs, as well as inputs make up circadian system. There are two levels that operates for this fundamental design: systemic and cellular. In case of systemic level, SCN orchestrates rhythmicity and the secretion of hormones. Molecularly, at cellular level, ubiquitous cell-autonomous oscillator contains interlinked positive and negative feedback loops. From ZT0 to ZT12 (day), CLOCK and BMAL1 bind to E-Box elements and act as transcription activators of the CRY and PER genes, whose products accumulate forming complexes that repress CLOCK-BMAL1 activity, which eventually inhibits their own expression [26]. In the evening, PER and CRY proteins dimerize with one another (associate with casein kinase 1δ (CK1δ) and CK1ε), translocate into nucleus, inhibiting CLOCK-BMAL1 transcriptional activity [27]. Ultimately, CRY and PER are polyubiquitinated by specific E3 ligase complexes (β-TrCP and FBXL3) for degradation [28], thus releasing the repression imposed by CLOCK-BMAL1 to restart cycle. From ZT12 to ZT0 (night), CLOCK and BMAL1 activate transcription of REV-ERBs and RORs, respective repressors and activators binding to ROR elements in BMAL1 promoter, enforcing rhythmic expression. By competitively binding ROR/REV-ERB response elements (ROREs), two nuclear receptor families – REV-ERBα/β and RORα/β/γ – stabilize core loop and induce an antiphase oscillation between PER2 as well as BMAL1 [29]. The repressor E4 promoter-binding protein 4 (E4BP4; also called NFIL3) and the basic leucine zipper (PAR-bZIP) proteins (TEF, DBP and HLF) that are rich in proline and acidic amino acids bind to D-boxes in a competitive manner resulting in a third type of transcriptional loop [7,30]. These loops, driven by CLOCK-BMAL1 or REV-ERB-ROR interactions, collectively regulate a wide array of target genes through cis elements (RORE, E-box and D-box) in promoters and enhancers (Figure 1). Altogether, these 14 transcriptional regulators produce the 24-hour rhythm.

*In vitro* studies in mouse embryonic fibroblasts show that the relative importance of negative (CRY/PER) and positive (CLOCK/BMAL1) complexes a key feature in generating rhythm and that PER could be a limiting factor in post-translational modifications (PTMs) [31]. Intestinal specific Bmal1 knockout in mice disrupted the liver’s ability to regulate blood sugar during fasting [32], showing that the liver’s internal clock helps anticipate daily changes in food intake by predicting nutrient availability. These findings indicate that molecular oscillators in peripheral clocks constitute a key factor in setting or adjusting the speed of the clock, which in turn commands metabolic signals.

## 3. Metabolic regulators

Biochemical processes exhibit rhythmic fluctuations that activate specific genes at the right time either during the day or night to convert nutrients into energy and sustain cellular metabolic functions. From past decades to the present day, locomotor activity monitoring and wrist actigraphy have been commonly used in mammals to quantify circadian rhythms [33,34]. Both methods record and analyse bodily (muscle) movement over time providing a behavioural proxy for circadian phase. Notably, skeletal muscle contraction itself serves as a peripheral zeitgeber that implies its direct effects in glucose metabolism [35,36]. This has been validated in transcriptomic studies which have demonstrated that there are approximately 1,000 rhythmic genes with a high amplitude. In particular, the genes revealed two apparent peaks of gene expressions: ZT4, associated with inflammation, immunity, and lipid metabolism, and ZT16, linked to muscle glucose metabolism and protein turnover [37]. These peaks are 12 h apart and they occur in opposite phases wherein one set of genes is active the other less active. This pattern reflects a well- orchestrated daily glucose uptake in muscles [38]. On a bigger scale, glucose limitation does not only obey the circadian rhythms, but also inhibits apoptosis through pyrimidine-dependent survival pathways [39,40]. These rhythms activate robust BMAL1 oscillations, influencing multiple signalling pathways, including Wnt/β-catenin, TGF-β/BMP, MAPK/ERK, NF-κB and Hedgehog pathways [41–45]. These routes are responsible in controlling the growth of chondrocytes and osteoblast replacement, which is important in bone formation. Interestingly, they also play crucial roles in muscle glucose metabolism by modulating insulin sensitivity, glucose uptake, and energy balance. Besides, the ubiquitous expression of circadian genes controlled by the SCN indicates the association between neural and metabolic activity. The initial recording of this relationship was done with the help of autoradiographic analysis of 2-deoxy-D-glucose (2-DG) uptake [46], which shows a high circadian fluctuation in SCN, the master clock of the brain that links neural activity to alterations in cerebral glucose activity.

According to the suggestion by Magistretti and Pellerin, glutamate is central in the stimulation of glycolysis in glial cells *via* a sodium-dependent postulate [47]. The release of glutamate is actively taken up by astrocytes using special glutamate transporters that also take sodium. This sodium influx triggers Na +/K + ATPase that subsequently stimulates glycolysis and glucose intake. Glycolytic product, lactate, is not broken down in astrocytes but rather transmitted to neurons as an energy source by monocarboxylate transporters. Remarkably, both the fly and mammalian version of the MCT-like gene mRNA transcript demonstrates cyclical expression indicating an expanded association of circadian rhythms with lactate transport [48,49]. The equilibrium between reduced and oxidated nicotinamide adenine dinucleotide (NAD) cofactors has a significant impact on cellular metabolic condition that modulates circadian rhythms as well as metabolism. The circadian clock is activated by NADH and inhibited by NAD+, with CRY proteins facilitating its conversion of NADH to NAD+, thereby influencing salvage pathway. *Invitro* studies show that the DNA-binding ability of CLOCK:BMAL1 and NPAS2:BMAL1 heterodimers improves in the presence of NADH and NADPH, while their oxidized forms inhibit binding [50]. It is worth noting that a minor change in the balance of NADH:NAD+ (60:40 to 80:20) leads to a switch between minimal and nearly maximum DNA-binding activity [51], suggesting that even subtle changes in the metabolic system can produce an effect on circadian gene regulation and cellular rhythm maintenance which is significant. It was shown that lactate directly activates NPAS2:BMAL1-dependent gene expression in cultured neuroblastoma cells [52] significantly reducing NAD+/NADH ratio *via* lactate dehydrogenase (LDH), which in turn influences NADP+/NADPH levels *via* nicotinamide nucleotide transhydrogenase (NNT). Further analysis of genomic studies in flies have identified the *Zw* gene, encoding glucose-6-phosphate1-dehydrogenase (G6PD), as a circadian-regulated gene [53]. G6PD catalyses rate-limiting step in pentose-phosphate pathway, generating reduced nicotinamide nucleotides, its rhythmic expression is likely to contribute to the circadian modulation of CLOCK:BMAL1 activity.

A substantial number of genes in liver, particularly those involved in metabolism, are under circadian regulation. These are genes that encode cytochrome P450, mitochondrial activity and heme biosynthesis enzymes [54]. Notably, rhythmicity is observed in a great number of transcripts in LD and not in DD conditions, indicating dependence on external cues rather than endogenous clocks. Among these cues, feeding stands out which has been shown to reset the circadian phase in the liver almost instantaneously, whereas altered light-dark conditions do not trigger a comparable change even after days [55]. This demonstrates the overriding mechanism of feeding rather than light in hepatic circadian rhythm synchrony. The circadian control of metabolic genes and pathways is supported by a number of experiments. An example is the uptake of glucose in SCN and other parts of the brain are rhythmic with the light-dark cycle. Also, genes that code for energy transport systems and metabolic enzymes such as glycogen phosphorylase, cytochrome oxidase, LDH and monocarboxylate transporter 2 (MCT2) exhibit circadian expression patterns [56].

A study investigating the impacts of metabolic injections on the circadian phase shifts in mice maintained in complete darkness, it was found that administration of 2-DG (500 mg/kg) resulted in the largest phase delay (∼90 min), whereas glucose (2 g/kg) caused a smaller delay [57]. Interestingly, when both 2-DG and glucose were administered together, the phase delay was reduced, suggesting that glucose counteracts the effects of 2-DG on circadian rhythms. These data prove the hypothesis that circadian phase shifts directly depend on the metabolic state. One intriguing phenomenon in circadian biology is the existence of a ‘dead zone’ a period during which the circadian clock exhibits minimal responsiveness to external stimuli. *In vivo* test indicates that exposure to light has an insignificant impact on circadian clock at ZT (4–8) [58]. Additionally, *in situ* hybridization studies confirm that Per gene expression in the SCN peaks during ZT (4–8) [59], explaining the clock’s insensitivity to light during this period. Nevertheless, light exposure between ZT (8–20) results in phase delays, while exposure between ZT (20–4) leads to phase advances. These shifts correspond with the activation of Per genes, which regulate circadian rhythms. This idea is also confirmed by experiments on phase response curve (PRC), where brief light pulses induce shifts in activity patterns. The steep induction curve of CLOCK:BMAL1 activity suggests that unless the NAD^+^/NADH ratio crosses a critical threshold [51,60], reduced NAD cofactor enrichment fails to activate transcription resulting in a redox insensitive ‘dead zone’. During this phase, reduced NAD cofactor levels are relatively low, diminishing DNA-binding activity. However, redox balance can be shifted towards reduced state by photic stimuli or neuronal activity enhancing CLOCK:BMAL1 DNA-binding thereby inducing phase shifts.

**Figure 2.**
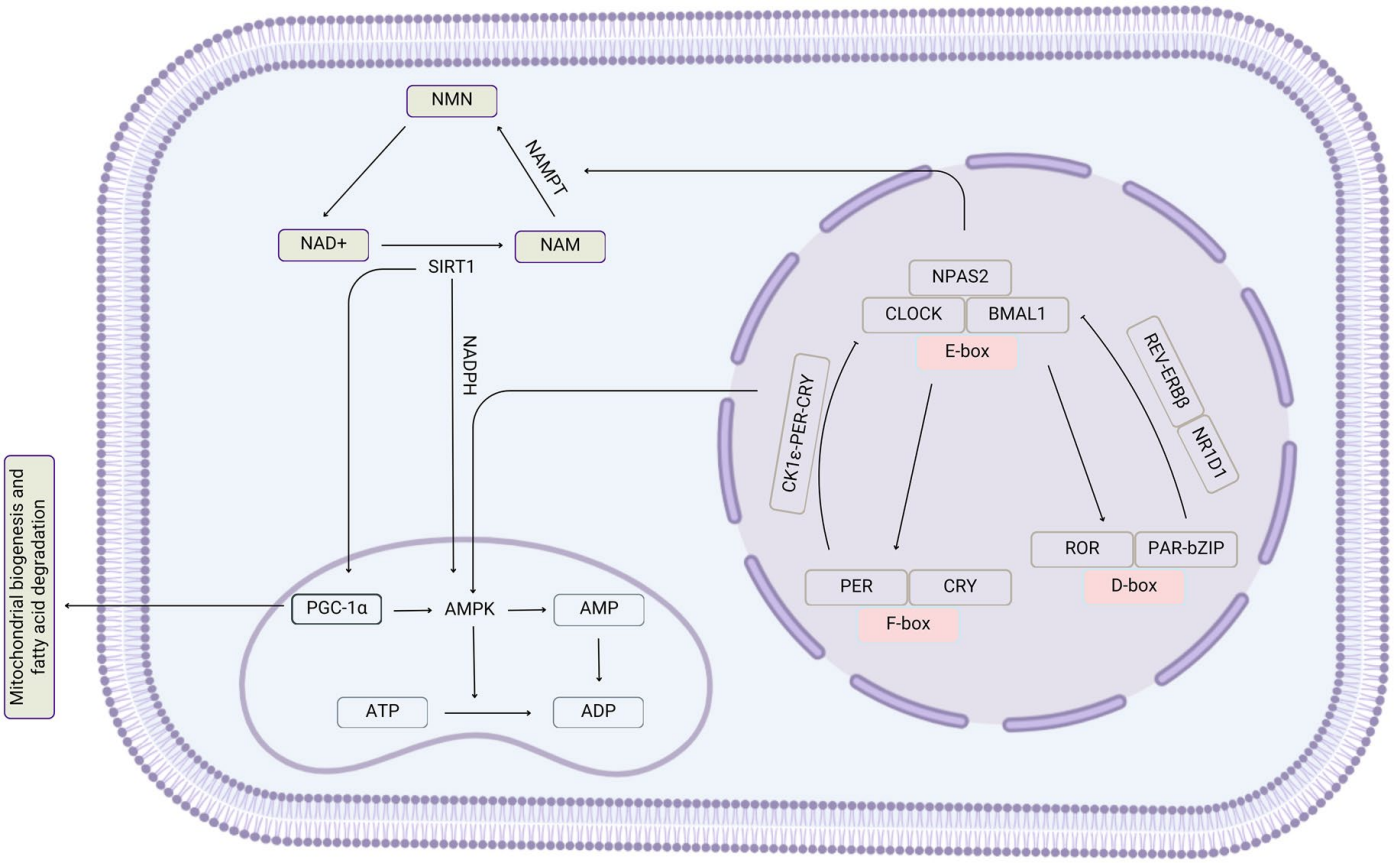
CLOCK:BMAL1 activity is gated by NAD^+^/NADH balance, with a saturation threshold forming a redox-insensitive ‘dead zone’. Photic and neuronal stimuli can shift redox ratios to re-engage DNA-binding and induce phase shifts. Beyond this zone, metabolic sensors AMPK and SIRT1 regulate clock dynamics *via* CRY1 turnover and CLOCK deacetylation. PGC-1α integrates these signals, requiring AMPK phosphorylation and SIRT1 deacetylation to coactivate BMAL1, forming a metabolic feedback loop essential for rhythmic gene expression.

Beyond the dead zone, circadian regulation is influenced by metabolic sensors such as Sirtuin 1 (SIRT1) and AMP-activated protein kinase (AMPK). SIRT1 acetylates CLOCK, regulating transcription cycles and linking the circadian clock to glucose homeostasis and energy metabolism. In the meantime, AMPK phosphorylates CRY1 marking it for degradation and modulating circadian transcript amplitudes while its loss stabilizes CRY1 and disrupts REV-ERBα expression [61,62], impairing circadian control (Figure 2).

### 4. Sirtuins

External changes are detected at the input pathway and converted to neural signals that are transmitted to central pacemaker for producing molecular oscillation of clock genes which in turn send signal to regulate physiological process such as mitochondrial biogenesis and cell apoptosis. These processes are achieved by post transcriptional alteration and degradation of circadian clock proteins by several enzymes among them sirtuins a class of nicotinamide adenine dinucleotide (NAD+) dependent histone deacetylases (HDACs) which catalyse deacetylation, ADP-ribosylation and chromatin remodelling to regulate transcriptional activity and cellular homeostasis [63]. In contrast to conservative HDACs, sirtuins are dependent on NAD +, which connects their function directly to cellular metabolic status. Each of the seven sirtuins are localized in different subcellular localizations and functions: SIRT1 and SIRT2 are found in the nucleus and cytoplasm and are involved in metabolism and stress response; SIRT3, SIRT4 and SIRT5 are mitochondrial sirtuins involved in metabolic regulation, oxidative stress and apoptosis; and SIRT6 and SIRT7 are nuclear sirtuins that regulate chromatin remodelling and DNA repair. Some acetylation procedures regulate circadian rhythm, SIRT1 overexpression in HEK293 cells suppressed CLOCK:BMAL1-driven Per1/Per2 expression [64], whereas its liver-specific deletion induced the acetylation of BMAL1 and the oscillation of NAD+ [65]. In addition, the periodical expression of Bmal1, Per2 and Dbp was disrupted by the knockout of SIRT1 in mouse embryonic fibroblasts (MEFs) and subsequently caused an increase in the protein levels of PER2 and CRY1 that control the histone and BMAL1 acetylation [66]. Similarly, SIRT6 knockout in HEK293 cells reduced PER2 stability while increasing its acetylation, and liver knockout altered the circadian transcriptome by increased BMAL1 chromatin binding, and H3K9 acetylation impacted fatty acid metabolism [67]. Meanwhile, the SIRT7 knockout in the mouse liver advanced the circadian phase and elevated CRY1 acetylation, which is also a sign of its impact on circadian regulation [68]. These results show their strong connection in aligning metabolism with circadian phases, while SIRT4, SIRT5 and SIRT6 may be potentially involved in tumourigenesis through circadian regulation [69,70]. SIRT4 suppresses the secretion of insulin and activates DNA damage response affecting cellular proliferation. SIRT5 controls apoptosis and oxidative stress, which potentially affects circadian rhythm disruptions in tumour cells. SIRT6 is a regulator of chromatin accessibility of circadian genes, that affect metabolism and oncogenic pathways [71,72]. Moreover, the glucose inhibition prevents the progress of NSCLC by activating the expression of Per *via* the AMPK-mediated upregulation of SIRT1 [73]. The essential reaction pathway of sirtuins is the hydrolysis of NAM to produce NAD+ that activates deacetylation of PGC-1α, which regulates mitochondrial biogenesis and metabolic adaptation through NAD+/NAMPT cycling [74], demonstrating their role in connecting metabolism and the circadian clock. The increase and decrease of NAD+ are regulated by NAMPT in a CLOCK-BMAL1-dependent way and SIRT1 promotes the transcription of NAMPT creating a feedback mechanism that maintains the circadian oscillation in NAD+. Then, the process of NPAS2 regulating the SIRT1 activity enhances the circadian regulation. Although NAMPT and NPAS2 interact indirectly *via* SIRT1, they may mediate their effect *via* independent yet complementary actions on circadian clock and metabolic functions. As discussed earlier feeding patterns are an effective zeitgeber of circadian rhythms, Calorie restriction with a moderate diet of 0.5% glucose enhances mitochondrial respiration and upregulates SIR2 activity [75].

The conversion of NAM to NAD+ is increased by NAMPT, which is regulated by the CLOCK-BMAL1-SIRT1 complex, thereby sustaining sirtuin activity [76]. The role of sirtuins in this process is highlighted by experimental evidence: Fibroblasts of SIRT1 knockout mice exhibit distorted BMAL1 and PER2 expression, which disrupt circadian rhythms [77], while SIRT6-deficient mice display altered lipid metabolism, which connects sirtuins to circadian activity under the influence of feeding [67]. SIRT1-overexpressing mice are leaner, more glucose-tolerant, and show improved metabolic health where null mice had elevated phosphorylation after 24 hr fast [78].

## 5. AMPK

Beyond the TTFL, post transcriptional modification integrates metabolic signalling, as systematic observations have documented that a significant subset of oscillating proteins doesn’t exhibit rhythmicity at mRNA level [79]. A key sensor in metabolic signalling is the heteromeric protein complex known as ‘adenosine monophosphate’ (AMP)–’activated protein kinase’ (AMPK), which is made up of catalytic α and regulatory β as well as γ subunits. Serine/threonine protein kinase and glycogen binding domain (GBD) are respective N-terminal of α and β subunits where complex formation is supported by C-terminal. The isoforms of α (α1, α2) and β (β1, β2) have less involvement compared to γ (γ1, γ2, γ3) on AMP/ATP binding due to the presence of CBS domain in C-terminal of γ subunit, collectively generating a cascade of cellular energy-sensing signals [80]. Under cellular stress ATP:ADP ratio declines causing high energy consumption, while Extracellular ATP exhibit rhythmic oscillations driven by the circadian clock [81]. A drop in ATP levels leads to the conversion of ADP to AMP, activating AMPK one of several metabolic sensors responsible for transmitting energy-dependent signals to initiate the catabolic pathway, finally restoring cellular balance. A key question to address is why AMPK is prioritized over other metabolic sensors. As part of efforts to explore this connection, researchers have identified amino acid sequence motifs that enhance AMPK-mediated phosphorylation [82,83], and evolutionarily conserved sequences in several clock components suggest preferential phosphorylation by AMPK [84]. This activation occurs only when muscle glycogen is depleted, allowing the GBD to localize AMPK to glycogen granules in the cytoplasm. AMPK senses glycogen depletion by interacting with peripheral oscillators in muscle and liver tissues [85], potentially influencing complex formation with the α and γ subunits by means of nutrient-dependent diurnal phosphorylation of CRY and PER proteins. Studies demonstrate that deleting the GBD disrupts this coordinated mechanism, impairing AMPK’s ability to respond to glycogen levels [86].

Regulatory protein modifications revealed that alanine; nonphosphorylatable amino acid, stabilizes CRY1 where aspartic acid; phosphomimetic amino acid, destabilizes it. Also CRY1 mutated by the replacement of serine at position 71 with aspartic acid reduces binding to PER2 while enhancing its interaction with Leucine-Rich Repeat Protein 3 (FBXL3), compromising its stability [87]. Additionally, the isoleucine to threonine (I364T) substitution and cysteine to serine (C358S) substitutions in Fbxl3 extends the circadian period in mice around 2–3 h[88,89]. This extension may be a chance of extended transition between the light and dark phase peaked by phosphorylation of per2 at Serine 394 [90]. These factors anchor FBXL3 role in ubiquitination of CRY1, primed by AMPK-mediated phosphorylation.

Casein Kinase I (CK1) is a unique group in serine/threonine protein kinase family has been identified as gene encoding *tau* locus [91], with its isoforms (α,β,γ1,γ2,γ3,δ and ε) being ubiquitously expressed in eukaryotes. Natural tau mutation In *Mesocricetus auratus* resulted in extended circadian period [92]. In addition, genetic disruption of CK1δ/CK1ε alters cellular and behavioural circadian rhythms [93]. AMPK -mediated activation of CK1ε phosphorylates several serin residues in PER2 located within negative amino acid sequence motifs determining PER stability and circadian period [94]. Genetic studies on *Drosophila sp.* double-time (dbt) prove orthologs of CK1δ/ε are required for normal phosphorylation and turnover of *dPER*. Interestingly, mutations in CK1δ caused more severe phenotypes compared to CK1ε, suggesting these kinases regulate PER2 degradation through distinct pathways. Although mutations in CK1ε or CK1δ, including null allele significantly impair circadian period in both mammals and drosophila but can’t abolish rhythmicity [95–97]. This is reinforced by data showing that CRY1 mutations at S71 or S280 do not affect stability, demonstrating the superiority of external cues in maintaining circadian rhythms [98]. Binding domains and phosphorylation sites of PER in mammals have been discovered, with CK1δ/ε-mediated phosphorylation inducing conformational changes. CRY forms a complex with PER and CK1δ/ε, preventing PER degradation and promoting the nuclear accumulation of the CRY–PER–CK1δ/ε complex [99]. This permits the transcription of CRY and PER by inhibiting the BMAL1–CLOCK complex’s transcriptional activity. Furthermore, under glucose restriction AMPK can suppressor NSCLC progression by upregulating BMAL1 [100]. Whether these PTMs are ubiquitous or tissue-specific, it results a noticeable alteration in running endurance, body weight, food habits and glucose homeostasis.

## 6. Dominance of feeding over light

High-fat diet (HFD), calorie restriction (CR), restricted feeding (RF) and intermittent fasting (IF) are different feeding patterns that have been shown to impact the circadian rhythm resetting to lower the occurrence of metabolic syndrome [101]. RF limits food availability to specific time without reducing caloric intake, uncoupling peripheral oscillators in liver and pancreas from SCN while leaving central pacemaker unaffected [102]. It enhances amplitude of clock gene expression promoting catabolic factor activity and reduces disease markers while improving overall health [103]. CR causes the caloric intake to drop to 60–75% of ad libitum concentrations, the effects of which directly affect the SCN, rearranging its time-of-day activity, enhancing light responsiveness, and synchronizing peripheral clocks [104]. The increased life span and decreased disease rates (age related) associated with IF are attributed to the fact that IF makes people more energy saving. It switches between unlimited feeding and starvation and the time of feeding that affects circadian rhythms [105]. In rodents daytime feeding disrupts liver clock genes, while nighttime feeding preserves rhythmicity. Improving glucose metabolism and stress resistance. HFD has an impact on peripheral clocks of hypothalamus, liver and the adipose tissue which delays the phases. Such feeding patterns also emphasize the overriding effect of timing of food on circadian regulation overcoming SCN [106]. Rhythmic feeding patterns entrain peripheral clocks, experimental studies show that even SCN-lesioned animals maintain robust circadian rhythm through food-entrainable oscillators (FEOs) [107,108]. Indicating the importance of the timing of feeding in alignment of physiological and behavioural mechanisms, demonstrating it’s capability of restoring metabolic dysfunction occurs as a result of derailed circadian rhythm. (Table 1).

**Table 1.** Clinical trials exploring feeding patterns as behavioural intervention in chronic metabolic disease management.

Interventions	Conditions	NCT number	Study status
Intermittent fasting	Prediabetes, Obesity	NCT06893913	Active
	Obesity	NCT06834256	Completed
	Polycystic Ovary Syndrome	NCT06804044	Completed
	Non-Alcoholic Fatty Liver Disease	NCT06666894	Completed
	NAFLD, Obesity	NCT06615817	Active
Calorie restriction	Non-Alcoholic Fatty Liver Disease	NCT06863376	Active
	Obesity	NCT06782009	Active
	Childhood Obesity	NCT06583447	Active
	Non-alcoholic Fatty Liver Disease	NCT05309642	Completed
	Non-Alcoholic Fatty Liver Disease	NCT03791203	Completed
High fat diet	Pre-diabetes	NCT05965973	Active
	Cystic Fibrosis-related Diabetes	NCT05766774	Active
	Obesity, Hypertension, T2DM	NCT04943926	Active
	Obesity, NAFLD, Type II Diabetes Mellitus	NCT04581421	Completed
Time restricted eating	Obesity	NCT06891352	Active
	Type 2 Diabetes	NCT06887543	Active
	Obesity	NCT06885255	Active
	NAFLD – Non-Alcoholic Fatty Liver Disease	NCT06705868	Active
	Obesity	NCT06616454	Active
	Metabolic Disease, Insulin Sensitivity	NCT06550115	Active
	Polycystic Ovary Syndrome	NCT06204965	Active
	Obesity	NCT05941871	Active
	Cardiovascular Health	NCT05628012	Active
	Type 2 Diabetes	NCT04155619	Active

## 7. Peripheral clocks and hormonal rhythmicity

The body’s internal clock regulates neurotransmitters both centrally and locally through the SCN. Serotonin, a key neurotransmitter involved in mood regulation and a primary target of most antidepressant drugs, exhibits rhythmicity. Circadian rhythm is ∼25 h but synchronized to 24-hour day-night cycle through light by retinal ganglion cells (ipRGCs) transmitting signal to SCN [109]. During darkness, transcription factors initiate melatonin secretion, which helps protect DNA and lipid molecules from oxidative damage by neutralizing reactive oxygen and nitrogen species, particularly hydroxyl radicals [110]. This antioxidant property is vital in tissues with high metabolic activity, like gastrointestinal tract (GIT) and brain. Nevertheless, exposure to ALAN disrupts this process. As a regulatory response, REV-ERBα functions antagonistically to Paired-like ETS transcription factor 1 (PET-1), nuclear activator of Tryptophan Hydroxylase 2 (TPH2) which catalyse rhythmic serotonin regulation [111]. Tryptophan is converted into 5-hydroxytryptophan (5-HTP) and subsequently decarboxylated into serotonin further undergoing acetylation and methylation to form melatonin, ensuring its availability for early-night regulation. Beyond serotonin biosynthesis, TPH1 and TPH2 exhibit tissue-specific expression, with TPH1 dominating adipose tissue where serotonin and kynurenine pathways intersect [112]. Kynurenic acid, a metabolite of kynurenine produced during exercise, promotes browning of white adipose tissue (WAT) enhancing mitochondrial function. Increased TPH2 expression in brain signals a protective response against mitochondrial dysfunction.

Reduced melatonin synthesis, particularly of MT1 subtype weakens antioxidant defence mechanisms interacting with nuclear retinoid receptors leading to insulin resistance and ROS-induced mitochondrial dysfunction [113]. Gastrointestinal tract (GIT) within Enteroendocrine (EE) cells synthesize and release melatonin in response to tryptophan-rich meals. Unfortunately, peripheral serotonin exerts effects in multiple metabolic tissues to promote nutrient absorption and storage, dampening natural metabolism. Therefore, inhibiting peripheral serotonin synthesis or signalling may be effective for treating obesity, T2DM, and non-alcoholic fatty liver disease [114]. Exogenous melatonin supplementation in chronotherapy has shown therapeutic benefits in conditions such as non-alcoholic steatohepatitis (NASH) by reducing liver enzyme levels as well as proinflammatory cytokines [115] and proven effective in resynchronizing ALAN disrupted circadian rhythms. GIT-derived melatonin contributes to circulating levels and provides protection for liver, biliary tract and stress-induced gastric lesions demonstrating its gastroprotective effects. Serum concentrations, measured *via* radioimmunoassay, show a strong correlation (*r* = 0.76) with nocturnal plasma concentrations when assessed alongside urinary 6-sulfatoxymelatonin (aMT6s) [116]. Since melatonin synthesis is directly influenced by light, controlled light exposure studies and chronotherapy underscore its potential as a diagnostic marker for circadian disorders.

The above discussion highlights that the circadian rhythm dictates the timing of multiple physiological activities and communicates with the body through a bidirectional process rather than a one-way interaction (Table 2). However, maintaining the sequential order of these activities depends on the robustness of individual’s rhythm.

**Table 2.** Hormones regulated by circadian clock.

Hormone	Mediator	Clock target	Reference
Melatonin	Aanat	Clock	[117]
Corticosterone	HPA axis dysregulation	Per2	[118]
Insulin	UCP2	Bmal1	[119]
Leptin	SOCS3 and FABP7	REV-ERBs	[120]
Ghrelin	GOAT	Bmal1	[121]
Serotonin	TPH2	REV-ERBα	[111]
Dopamine	Tyrosine hydroxylase	REV-ERBα	[122]
Testosterone	PKA-StAR, HSD17B3	Bmal1, Per1/Per2	[123,124]
Oestrogen	ERα, Erβ	Clock, Bmal1	[125]
Prolactin	Prolactin receptor	Bmal1	[126]
Triiodothyronine, thyroxine	OATP1C1 and DIO2	Bmal1, Cry1	[127,128]
Aldosterone	Epithelial Na + channel, HSD3B2	Cry1/2 Per1	[129,130]
Oxytocin	binds PVN target genes	Bmal1	[131]
Growth Hormone	GH/GHR signalling	Clock, Bmal1	[132,133]

## 8. Benefits of targeting circadian rhythm

Most people either have a sleep-wake cycle or feeding-fast cycle influenced by circadian rhythm which peaks during first meal, last meal, wakeup and sleep. Targeting these timing with drug delivery minimize side effect and maximize effectiveness, in type 2 diabetes Sodium-Glucose Cotransporter 2 (SGLT2) inhibitors administered at night induced improved glucose control [134]. Researchers using melatonin to treat insomnia, documented good sleep quality and stable circadian rhythm. This strategy aligns medicine administration with biological rhythm. Switching from long half-life drugs to short half-life drugs restores daily rhythm. Additionally, targeting the circadian rhythm in cancer therapy has shown synergistic effects with commonly used drugs such as doxorubicin and cisplatin [135–137]. More than 80% of FDA approved drug targets exhibit daily rhythm in mRNA level [138,139]. Statistics predict that the prevalence of diabetes and cancer will increase in upcoming years [140]. Therefore, even small improvements in these drugs could have a significant impact on a large population. The evidence outlined earlier, alongside pharmacological interventions such as treatments with forskolin, phorbol-12-myristate-13-acetate (PMA), and calcimycin, alters circadian gene expression *via* distinct pathways [141–143]. Despite their differences, all these treatments synchronize a stable circadian oscillation, emphasizing the robustness of the circadian control system. These findings highlight a fundamental principle: different drugs, acting through different pathways, initially exerts effect on circadian gene expression, followed by a drug-specific response.

Adipocytes from healthy subcutaneous fat sample had increased cellular insulin sensitivity by ∼30% when compared to those from sleep-deprived individuals [144]. This reduction is primarily due to excessive adipogenesis caused by loss of control over PPARγ in differentiating pre-adipocytes into mature adipocytes also leading to hypertrophy (enlarged adipocytes) and hyperplasia (increased adipocyte numbers). Hypertrophic adipocytes are less insulin-sensitive and secrete pro-inflammatory cytokines such as TNF-α and IL-6, which further disrupt insulin signalling pathways. These impaired adipose tissue results in the development of metabolic syndrome and T2DM.

Pre-clinical and controlled laboratory studies revealed that clock components regulate several oncological and metabolic signalling pathways. Investigating the effects of resveratrol on high-fat diet-induced circadian disruption in C57BL/6 mice showed that rhythmic glucose and lipid metabolism was restored by modulating Clock, Bmal1, Per2 and Sirt1 [145], highlighting its potential to mitigate metabolic disorders *via* circadian regulation. Individuals exposed to shift work or jet lag experience disrupted circadian rhythms, with just 4–5 days of misalignment significantly increasing 2-hour postprandial glucose levels (plasma glucose >8.06 mmol/l) classifying them as prediabetic confirmed by reduced aMT6s, which is positively associated with an increased risk of diabetes [146].

Melatonin targeting circadian rhythm through Gi-coupled MT2 receptors in pancreatic β-cells reduces intracellular calcium level and oxidative stress in rodent β-cell lines [147] (INS-1 832/13) and human islets from type 2 diabetic patients, with ramelteon treatment Bmal1 and Rev-erbα mRNA expression are enhanced in rat INS-1 cells [148]. Experiments by Reiter and colleagues showed that injecting alloxan or streptozotocin, which destroy β-cells and induce diabetes, abolished the nighttime increase in melatonin in Syrian hamsters (*Mesocricetus auratus*) [149]. This disruption occurs because the circadian rhythms of MT1/MT2 and RORα receptors depend on serum and intracellular melatonin levels. These disruptions can be corrected by melatonin agonist Neu-P11 (piromelatine) [150] which inhibits weight gain, improves insulin sensitivity as well as glycaemic control and reduces HbA1c levels in diabetic patients. Serum glucose levels in rat models of diabetes were also normalized after 6 weeks of melatonin treatment at a concentration of 10 mg/100 ml in drinking water [151].

## 9. Chronotherapy

Chronobiology is a broad field of biology that studies biological phenomena exhibiting cyclical behaviour. An important application of this field is chronotherapy, which involves targeting drug release at specific times of the day to maximize clinical effectiveness. Emphasizing oscillatory signatures and selective chromatin remodelling of clock-controlled genes (CCGs) helps identify rhythmic markers [152], optimizing dosing times for improved drug diffusion and efficacy. Oscillating protein carrier levels instructs precise treatment strategies, guiding time-delayed drug release to develop safer and more effective medications. When these oscillations function normally, they support chronophysiology by determining the optimal timing for drug administration based on the dynamic behaviour of proteins. Another critical aspect is xenobiotic metabolism and detoxification, key factors influencing drug efficacy and toxicity which exhibit daily oscillations across the gastrointestinal, hepatic and renal systems [153]. Multiple oscillations of rate-limiting regulatory enzymes in essential metabolic pathways establish optimal treatment windows for enhancing therapeutic outcomes. These chrono pharmacological changes have an impact on pharmacokinetics and pharmacodynamics. Chronopathology, the science of abnormal or perturbed biological rhythms expounds on how these oscillations alter receptor availability, secondary messenger systems, metabolic pathways and drug-protein interactions. The information has given rise to a chronotherapeutic drug delivery system (CDDS), which are designed to match the natural rhythms of the targets to enhance the therapeutic response of the body. These systems work using various mechanisms to optimize the accuracy of treatment. Some are time-programmed, where drugs are delivered at specific intervals in order to synchronize with disease rhythms. Others are internally provoked, reacting to the changes in the body such as, temperature-sensitive carriers in managing fever or glucose-sensitive formulations in managing insulin release in diabetes. Moreover, the externally stimulated systems utilize stimulating attempts like electric surgeries, magnetic fields, or MEMS wireless devices to allow on-command drug modulation [154].

**Figure 3.**
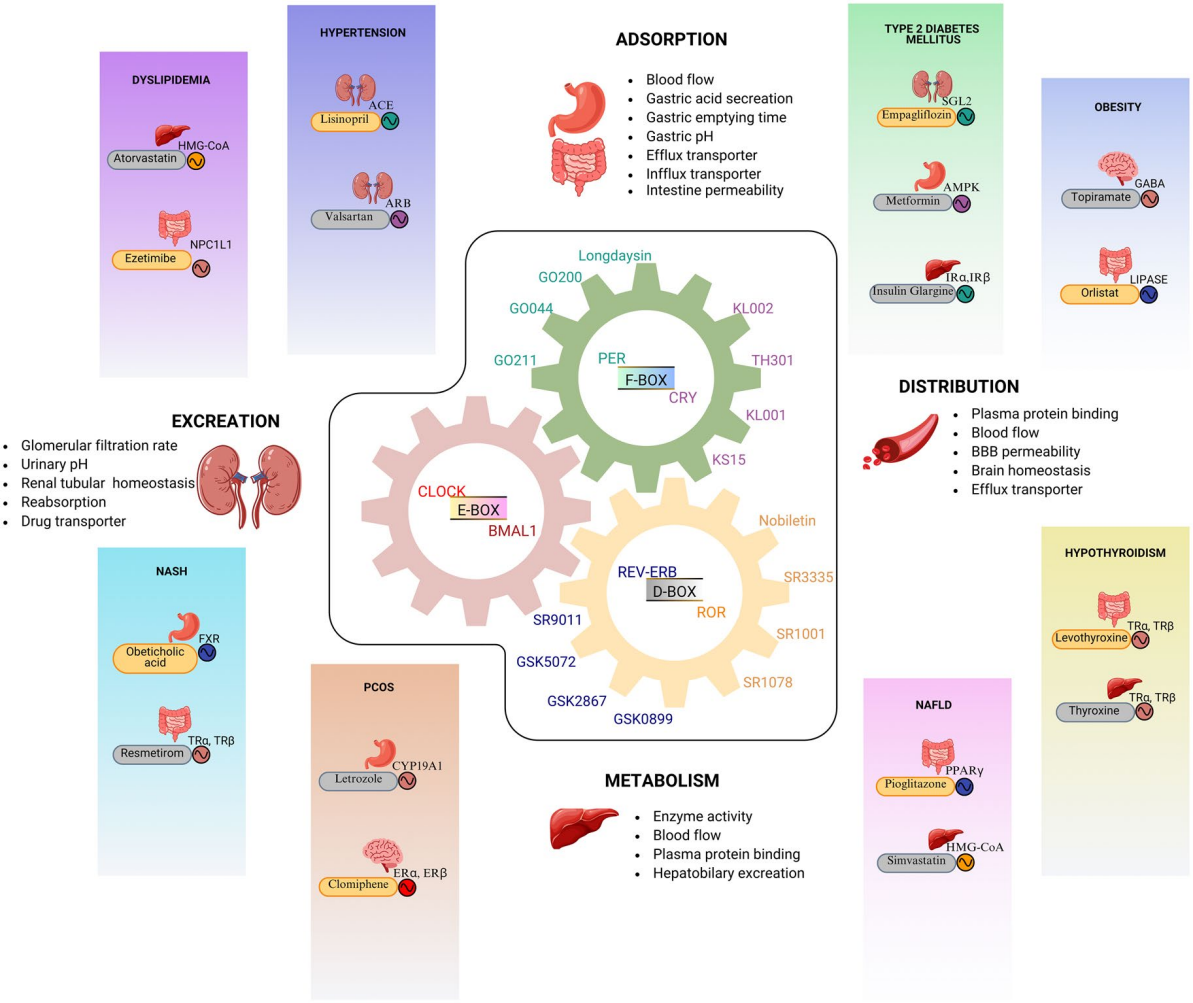
Illustrates the interplay between circadian clock regulatory elements and drug (ADME) administration, distribution, metabolism and excretion. Drugs are used to maintain robust expression of clock genes, which in turn regulate these pharmacokinetic processes. Target receptors for chronic metabolic disease are shown to be regulated by clock genes therefore drugs administration in time-dependent (day/night) manner optimize therapeutic outcomes. Emphasizing the critical role of circadian rhythms in chronotherapy for pharmacological effectiveness.

The traditional dosage types are usually ineffective in combating diseases with a chrono pathological basis, whereas CDDS effectively mimic circadian rhythms thus optimizing drug pharmacokinetics and optimizing treatment outcomes (Figure 3). The gut clock regulates the absorption of drugs by modulating gastric pH, motility and expression of transporters [155]. Lipophilic drugs such as Propranolol [156] and Simvastatin [157] show high absorption in the morning because of enhanced gut perfusion, but water-soluble drugs show stable absorption throughout the day. Drug distribution varies with fluctuations in blood flow, plasma protein levels and transporters, leading to peak distribution of drugs like Warfarin and Morphine during the active (daytime) phase, increasing their therapeutic response. Most liver’s metabolic enzymes, particularly cytochrome P450 is under circadian regulation tempering the rates of drug metabolism. For example, Paracetamol is most efficiently metabolized at night [158], while corticosteroids like Prednisone exhibit peak metabolism in the morning Similarly, drug excretion occurs in a rhythmic fashion because renal blood flow and filtration rates fluctuate throughout the day, which has an optimal effect on the elimination of drugs like Furosemide and Lithium in the morning [159]. Reduced oxaliplatin tolerability in CRY1/CRY2 knockout models, further underscore the role of clock genes in renal drug clearance [160]. With all the immense chrono pharmacological variations, the integration of circadian rhythms in clinical practice is becoming an emerging priority. This change received strong impetus following the groundbreaking discovery of the molecular mechanisms governing the intrinsic circadian pathway recognized with the 2017 Nobel Prize in Physiology or Medicine, awarded to Jeffrey C. Hall, Michael Rosbash and Michael W. Young. Since then, there has been a growing adaptation in the incorporation of circadian-based treatments in clinical research. As of 2016, there were 217,258 registered clinical trials [161], with only 348 incorporating circadian interventions or chronotherapy. However, by 2025, this number has more than doubled to 515,000 trials, with 1,101 now integrating chronotherapy. A statistical z-test confirmed an extremely significant p-value (<0.01), underscoring the growing recognition of circadian rhythms as a critical factor in therapeutic strategies.

## 10. Conclusion

Metabolic homeostasis is largely influenced by circadian rhythms, which regulate gene expression, hormonal pathways and nutrient metabolism. Precise timing of metabolic processes is ensured by the interaction between central circadian clocks in the SCN and peripheral clocks, which guarantees physiological homeostasis. Organ systems may function independently, but their functioning is very much interdependent and controlled temporally to avoid disruption and to maximize efficiency. Systemic coordination of metabolic, endocrine and cardiovascular functions, occurs through the rhythmical organization of the circadian clock to maintain a coherent system response to external stimuli, and to maintain homeostasis. These rhythms are disrupted by exposure to ALAN, irregular feeding patterns and contemporary lifestyles, leading to metabolic disorders such as obesity and diabetes. Molecular research into the mechanisms of circadian regulation has shown that metabolic sensors such as SIRTs and AMPK are important for energy balance and cellular health. Dysfunction of these pathways do not only contribute to impaired metabolic flexibility but also significantly increase the risk of developing chronic diseases and thus represent an important area of intervention. Implementing chronobiology in clinical settings through chronotherapeutic drug administration systems, personalized treatments based on a disease’s chronopathology, and adjusting drug administration according to chronopharmacokinetics and chronopharmacodynamics can result in targeted control over circadian regulators, leading to enhanced metabolic resilience and improved therapeutic outcomes.

## 11. Future directions

Consideration of circadian biology as a medical practice is experiencing increased attention, with prospects of enhancing treatment efficacy and metabolic well-being. Future studies ought to aim optimizing chronotherapeutic measures and audit therapies that restore circadian integrity in subjects using rhythm-based therapies. There is an ever-growing realization that the organ-specific therapies are becoming obsolete when considering body’s interconnected circadian regulation. As an example, melatonin boosts circadian activity and suppresses β-cell apoptosis, and metformin increases insulin sensitivity but does not have a direct impact on circadian activity. Nevertheless, a combination of their use proved to have the synergistic effect as in the case of live animal studies of the insulin-producing cell function and morphology [162]. Resting on this premise, the future of precision medicine is in customizing the treatment in terms of the genetic and circadian profile of an individual. Circadian cycles are unique among individuals, and thus optimal medication timing is crucial for maximum efficacy, since most of the drugs sold in the market are directed towards genes that have periodically fluctuating expression. Nevertheless, as standard medical practice has been largely ignored, the variations have been neglected because there has been a limited mechanistic knowledge about them. Positively, a large number of pharmaceutical firms are currently stocking up in circadian-based drugs development to improve drug effectiveness. However, a major disadvantage is that much of historical research has been based on nocturnal animal experimental models which are not always compatible with the diurnal human body. There is also the possibility that some drugs with highest efficacy in animals do not have the same effect in humans at the same time creating a necessity of using species-specific circadian considerations in clinical trial. The benefit of resolving these variations will be in the active enhancement of treatment regimens and the development of a personalized, circadian-based treatment, which will enhance therapeutic efficacy.

## Data Availability

No data were created or analyzed in this study.

## Abbreviations

TTFLs: Transcription-Translation Feedback Loops
SCN: Suprachiasmatic Nucleus
AMPK: AMP-activated protein kinase
SIRTs: Sirtuins
ALAN: Artificial Light at Night
CCGs: Clock-Controlled Genes
CR: Circadian Rhythm
CLOCK: Circadian Locomotor Output Cycles Kaput
BMAL1: Brain and Muscle Arnt-Like Protein 1
CRY: Cryptochrome
PER: Period
CK1: casein kinase 1
FBXL3: F-Box and Leucine-Rich Repeat Protein 3
RORs: Retinoic Acid-Related Orphan Receptors
ZT: Zeitgeber Time
ipRGCs: retinal ganglion cells
CBS: Cystathionine Beta-Synthase
NAD: Nicotinamide Adenine Dinucleotide
NMN: Nicotinamide Mononucleotide
ATP: Adenosine Triphosphate
ADP: Adenosine Diphosphate
NPAS2: Neuronal PAS Domain Protein 2
NNT: Nicotinamide Nucleotide Transhydrogenase
NAM: Nicotinamide
GBD: Glycogen Binding Domain
PGC-1α: Peroxisome Proliferator-Activated Receptor Gamma Coactivator 1-Alpha
2-DG: 2-Deoxy-D-glucose
LDH: Lactate Dehydrogenase
ARV: Arcuate Nucleus
PVN: Paraventricular Nucleus
E4BP4: E4 Promoter-Binding Protein 4
PAR-bZIP: Proline and Acidic Amino Acid-Rich Basic Leucine Zipper
TEF: Thyrotroph Embryonic Factor
DBP: D-site Binding Protein
HLF: Hepatic Leukaemia Factor
TGF-β: Transforming Growth Factor-Beta
BMP: Bone Morphogenetic Protein
MAPK: Mitogen-Activated Protein Kinase
ERK: Extracellular Signal-Regulated Kinase
NF-κB: Nuclear Factor Kappa-Light-Chain-Enhancer of Activated B Cells
G6PD: Glucose-6-Phosphate 1-Dehydrogenase
MCT2: Monocarboxylate Transporter 2
GIT: Gastrointestinal Tract
PET-1: Paired-like Homeodomain 1
TPH2: Tryptophan
5-HTP: 5-Hydroxytryptophan
NASH: Non-Alcoholic Steatohepatitis
aMT6s: 6-Sulfatoxymelatonin
SGLT2: Sodium-Glucose Cotransporter 2
PPARγ: Peroxisome Proliferator-Activated Receptor Gamma
TNF-α: Tumor Necrosis Factor-Alpha
INS: Insulin
IL-6: Interleukin-6
MT1: Melatonin Receptor 1
HbA1c: Glycated Haemoglobin
UCP2: Uncoupling Protein 2
SOCS3: Suppressor of Cytokine Signalling 3
FABP7: Fatty Acid Binding Protein 7
DIO2: Type II Iodothyronine Deiodinase
GOAT: Ghrelin O-Acyltransferase
OATP1C1: Organic Anion Transporting Polypeptide 1C1
